# Emission Enhancement of ZnO Thin Films in Ultraviolet Wavelength Region Using Au Nano-Hemisphere on Al Mirror Structures

**DOI:** 10.3390/nano15050400

**Published:** 2025-03-06

**Authors:** Shogo Tokimori, Kai Funato, Kenji Wada, Tetsuya Matsuyama, Koichi Okamoto

**Affiliations:** 1 Department of Physics and Electronics, Graduate School of Engineering, Osaka Metropolitan University, 1-1 Gakuen-cho, Naka-ku, Sakai 599-8531, Osaka, Japan; 2 Equipment Sharing Center for Advanced Research and Innovation, Osaka Metropolitan University, 1-1 Gakuen-cho, Naka-ku, Sakai 599-8531, Osaka, Japan

**Keywords:** plasmonics, ultraviolet, plasmon enhanced luminescence, zinc oxide

## Abstract

Using a heterogeneous metal Nano Hemisphere on Mirror (NHoM) structure, composed of an Al_2_O_3_ thin film and Au nano-hemispheres formed on a thick Al film, we successfully generated two distinct surface plasmon resonance (SPR) peaks: one in the ultraviolet (UV) wavelength range below 400 nm and another in the visible range between 600 and 700 nm. This NHoM structure can be fabricated through a straightforward process involving deposition, sputtering, and annealing, enabling rapid, large-area formation. By adjusting the thickness of the Al_2_O_3_ spacer layer in the NHoM structure, we precisely controlled the localized surface plasmon resonance (LSPR) wavelength, spanning a wide range from the UV to the visible spectrum. Through this tuning, we enhanced the band-edge UV emission of the ZnO thin film by a factor of 35. Temperature-dependent measurements of emission intensity revealed that the NHoM structure increased the internal quantum efficiency (IQE) of the ZnO thin film from 8% to 19%. The heterometallic NHoM structure proposed in this study enables wide-ranging control of SPR wavelengths and demonstrates significant potential for applications in enhancing luminescence in the deep ultraviolet (DUV) region, where luminescence efficiency is typically low.

## 1. Introduction

Surface plasmon resonance (SPR) is a phenomenon where collective oscillations of free electrons on a metal surface interact with an external electric field. When SPR is induced, the resonant light energy becomes confined to the metal-dielectric interface, leading to a significant electric field enhancement. Due to their exceptional optical properties, SPRs have been extensively studied across various fields, including sensing [1,2,3,4,5], imaging [6], solar cells [7,8], and the enhancement of light emission in semiconductor optical devices [9].

Ultraviolet (UV) light with wavelengths below 400 nm is highly significant for practical applications, including water and air purification as well as sterilization [10]. Unlike chemical disinfectants such as alcohol, UV disinfection does not cause material degradation, making it ideal for both industrial processes and daily use. In industrial applications, it is utilized in semiconductor lithography [11] and resin curing [12], while in medical applications, it is employed for the treatment of skin diseases such as vitiligo and psoriasis [13], hemodialysis [14], and in bioapplications such as quantitative protein analysis [15]. Given its broad range of applications, there is an increasing demand for highly efficient ultraviolet light sources. As a result, extensive research has been conducted to improve the luminescence efficiency of LEDs emitting in the UV to deep ultraviolet (DUV) wavelength range [16,17,18,19,20,21]. AlGaN-based LEDs are widely utilized as deep-ultraviolet light-emitting materials. However, their high threading dislocation density results in low internal quantum efficiency. Additionally, UV light absorption by the p-GaN contact layer and electrode further reduces light extraction efficiency. To address these challenges, extensive research has been conducted. Key advancements include enhancing internal quantum efficiency through the development of AlN buffer layers with low threading dislocation density [22] and improving light extraction efficiency using transparent AlGaN:Mg contact layers [23,24]. However, achieving even higher luminescence efficiency often requires more complex fabrication processes, significantly increasing production costs. To overcome these limitations, we aim to enhance luminescence efficiency using SPR through a cost-effective and straightforward approach.

The enhancement of luminescence efficiency using SPR was first reported in 2004 by Okamoto et al., who demonstrated a 14-fold enhancement in blue luminescence of InGaN quantum wells using Ag films [9]. This enhancement occurs when the energy of surface plasmons (SPs) matches the energy of excitons in the quantum wells, leading to the coupling between the SP and excitons. This SP–exciton coupling increases light emission efficiency by promoting spontaneous emission and suppressing non-radiative recombination.

To enhance luminescence efficiency in the DUV wavelength range using SPR, Al, which exhibits SPR across a wide range from the visible to the DUV range, is commonly employed [25]. In 2010, we demonstrated a 7-fold enhancement of DUV emission at a wavelength of 270 nm from AlGaN/GaN quantum wells by utilizing propagating SPR with Al thin films [26]. However, the resonance wavelength of propagating SPR is determined by the dielectric properties of the metal and the surrounding medium, making it difficult to tune the resonant wavelength. When the emission and SPR wavelengths are significantly mismatched, the enhancement effect is rapidly reduced. Localized surface plasmon resonance (LSPR) is considered a promising solution to this challenge. LSPR occurs when light interacts with metallic nanostructures smaller than the incident wavelength. Nanostructures such as nanoparticles [16,27], nanodisks [28,29,30], nanohole arrays [31,32], and nanovoids [33,34] are used to generate LSPR. By adjusting the size and shape of these nanostructures, the resonance wavelength can be finely tuned due to the delay effect in the electronic vibrations within the metal. Al nanostructures are particularly effective for generating LSPR in the UV range. For instance, a 33.3% enhancement of DUV light at a wavelength of 285 nm from AlGaN quantum wells was achieved using Al nanodisks, fabricated via electron beam lithography [16]. Additionally, a study reported a 2.4-fold enhancement of near-band-edge emission at 387 nm from ZnO using periodic Al nanoparticles [17]. However, producing large-area Al nanostructures using conventional nanofabrication techniques is time-consuming. Moreover, the enhancement is smaller than the previously mentioned 7-fold increase at 260 nm from AlGaN using an Al thin film [26], highlighting the need for stronger enhancement through nanostructures. An alternative approach involves forming nano-hemispherical structures by annealing metal thin films. However, when applying this hemispherical fabrication method to Al, controlling the size of the Al nano-hemispheres is more challenging compared to other plasmonic metals such as Ag and Au, due to the high adhesiveness of Al. Attempts to control the size of the Al nanospheres and the LSPR wavelength in the UV range have included enhancing the hydrophobicity of the substrate surface using chemicals [35], but controlling the resonant wavelength over a broad wavelength range remains difficult.

In 2020, we developed the Nano-Hemisphere on Mirror (NHoM) structure, which can be fabricated through a straightforward process involving deposition, sputtering, and heat treatment, and enables LSPR wavelength control [33]. The NHoM structure consists of a dielectric thin film deposited on a thick metal layer, with metallic nano-hemispheres formed on top, and can be fabricated without using complex nanofabrication techniques. The coupling of the SP modes of the nano-hemispheres and their mirror-image modes, induced in the underlying metal film, causes the splitting of the SP resonance modes, resulting in significant electric field enhancement. Using NHoM structures fabricated with Ag, we successfully generated LSPR in the visible wavelength range and achieved precise control of the resonant wavelength [36,37]. Resonances induced by films are represented as SPR, while those induced by nanostructures, including NHoM structures, are represented as LSPR. To achieve LSPR in the DUV wavelength range, NHoM structures were fabricated using Al. While LSPR was successfully generated, the uniform fabrication and size control of Al nano-hemispheres through heat treatment proved challenging, and the control range of the resonant wavelength did not extend across the entire UV spectrum [38]. To address this issue, we developed a hybrid material NHoM structure by combining Ga nano-hemispheres or Ga_2_O_3_ nano-hemispheres formed by heating the Ga nano-hemispheres, and a thick Al film. This structure successfully generated an LSPR peak at 200 nm in the DUV range without relying on Al nanostructures [39]. In NHoM structures composed of the same metal, the LSPR peak becomes sharper due to the coupling between the LSPR mode in the upper nano-hemisphere and the mirror-image mode induced in the thick metal film compared to a structure with metal nanospheres. In contrast, the Ga_2_O_3_/Al-NHoM structure, composed of different materials, does not generate LSPR in the dielectric Ga_2_O_3_ nano-hemisphere but induces the plasmon mode of underlying Al layer, enabling DUV LSPR generation even when using materials that do not inherently SPR in the DUV range.

In this study, we propose a novel heterometallic NHoM structure consisting of Au nano-hemispheres, an Al_2_O_3_ spacer, and an Al film, with the aim of enhancing the emission of ZnO that exhibits emission at a wavelength of 380 nm. Compared to Al, Au enables easier formation of uniform nano-hemispheres through heat treatment. By adjusting the thickness of the dielectric spacer and controlling the distance between the metal film and the nano-hemispheres, the LSPR wavelength can be tuned over a broad range. We experimentally verified the tunability of the LSPR wavelength by varying the thickness of the dielectric spacer and the size of the Au nano-hemispheres and analyzing the reflected extinction spectra. Furthermore, the luminescence enhancement effect and its underlying mechanism in the fabricated NHoM structures were investigated by depositing ZnO thin films onto the structures and conducting photoluminescence (PL) measurements, including the temperature dependence of PL intensity. Previous studies have demonstrated that NHoM structures achieve SPR peaks at wavelengths of approximately 500–700 nm [36,37] and 200 nm [38,39]. In this study, we aimed to control the wavelength within the 300–500 nm range using a heterometallic NHoM structure. Additionally, while Maeda et al. demonstrated the practicality of the NHoM structure as a plasmonic color sensor [37], this study presents, for the first time, its luminescence enhancement effect.

## 2. Materials and Methods

### 2.1. Calculations

**Figure 1:**
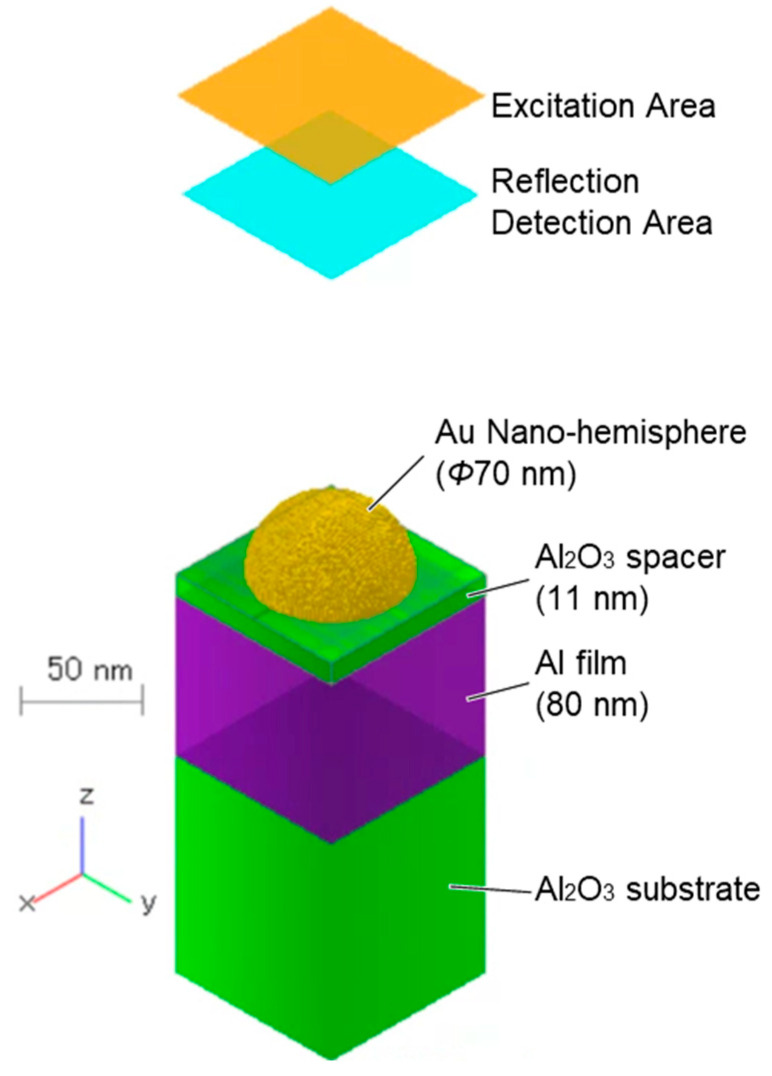
Schematic model of the NHoM structure used in the FDTD simulations.

Electromagnetic field analysis was conducted using the finite-difference time-domain (FDTD) method to investigate the SPR characteristics of the NHoM structure. The numerical simulations were performed using commercial software (Poynting for Optics V03L10R131, Fujitsu, Tokyo, Japan). The model of the NHoM structure used in the simulations is shown in Figure 1. The NHoM structure consists of an Al layer (purple) on an Al_2_O_3_ substrate (green), with metallic nano-hemispheres (yellow) placed on top via Al_2_O_3_ spacer layers. The diameter of the metal hemisphere was set to 70 nm, and the thickness of the Al_2_O_3_ spacer was set to 11 nm. For the simulations, periodic boundary conditions were applied in the X and Y directions, while absorption boundary conditions were applied in the Z direction. The excitation source was a light pulse with a width of 0.3 fs, an electric field strength of 1 V/m, a central frequency of 600 THz (corresponding to a wavelength of 500 nm), and X-axis polarization. The dielectric functions of Al and Au were modeled using the Lorentz–Drude model. Non-uniform meshes with grid sizes ranging from 0.5 nm to 2.0 nm were used for the simulations. The excitation pulses were irradiated from the top of the structure, and the reflection spectrum was observed in the reflection detection area. The reflected extinction spectrum, *E*, was then calculated using the following equation:

(1) $$E=-{log}_{10}\left(\frac{R}{R_{0}}\right)$$

where *R* represents the reflection spectrum of the NHoM structure sample, and *R*_0_ represents the reflection spectrum of the sample consisting of the Al film only.

### 2.2. Fabrication Method

All nanostructures were fabricated on (0001) sapphire substrates. The sapphire substrates were first ultrasonically cleaned in acetone for 5 min, followed by ultrasonic cleaning in ethanol for 10 min, then rinsed with ethanol and dried using compressed air. An 80 nm Al (purity 99.99%) film was deposited onto the cleaned sapphire substrates using a resistance heating evaporation system (SVC-700TMSG, Sanyu Electron, Tokyo, Japan). The chamber pressure before Al deposition was 1.0 × 10^−3^ Pa, and Al was deposited at a rate of 20 Å/s. Subsequently, Al_2_O_3_ thin films, with thicknesses between 5 nm and 30 nm, were deposited using RF sputtering equipment (FRS-2CP-260T, FKD Factory, Tokyo, Japan) or atomic layer deposition system (SAL1000, SUGA, Hokkaido, Japan). The chamber pressure before Al_2_O_3_ deposition was 5.0 × 10^−4^ Pa, and Al_2_O_3_ was deposited at a rate of 0.01 Å/s. The Al_2_O_3_ target used had a purity of 99.99%. Au (purity 99.99%) thin films, with thicknesses between 3 nm and 18 nm, were then deposited by evaporation onto these layers. The chamber pressure before Au deposition was 1.0 × 10^−3^ Pa, and Au was deposited at a rate of 0.6 Å/s. The samples were annealed at 630 °C under a nitrogen atmosphere using an electric furnace (F0110, Yamato Scientific Co., Ltd., Tokyo, Japan) to fabricate the NHoM structures. The samples were heated in an electric furnace, which preheated to the set temperature, and removed immediately after 10 min.

### 2.3. Observations and Measurements

The sample surfaces were characterized using an atomic force microscope (AFM) (Nanocute, Hitachi High-Tech, Tokyo, Japan). Reflectance spectra were measured with a spectrophotometer (UV-2600, Shimadzu, Kyoto, Japan) and subsequently converted into extinction spectra. A ZnO thin film was then deposited onto the NHoM structures using RF sputtering equipment. The chamber pressure before ZnO deposition was 1.0 × 10^−3^ Pa, and ZnO was deposited at a rate of 0.5 Å/s. The ZnO target used had a purity of 99.99%. For PL measurements, a He-Cd laser (IK3202R-D, KIMMON, Tokyo, Japan) with a wavelength of 325 nm and a power of 200 mW was used as an excitation light. The ZnO luminescence spectra were measured using a multichannel spectrometer (PIXIS 100B-3, SpectraPro 2300i, Roper Scientific, Trenton, NJ, USA). The temperature dependence of the luminescence intensity was measured using a closed-cycle helium cryostat (CG-C-205DT, Nagase, Kanagawa, Japan), which enables precise temperature control in the range of 8 K to 300 K.

## 3. Results and Discussion

**Figure 2:**
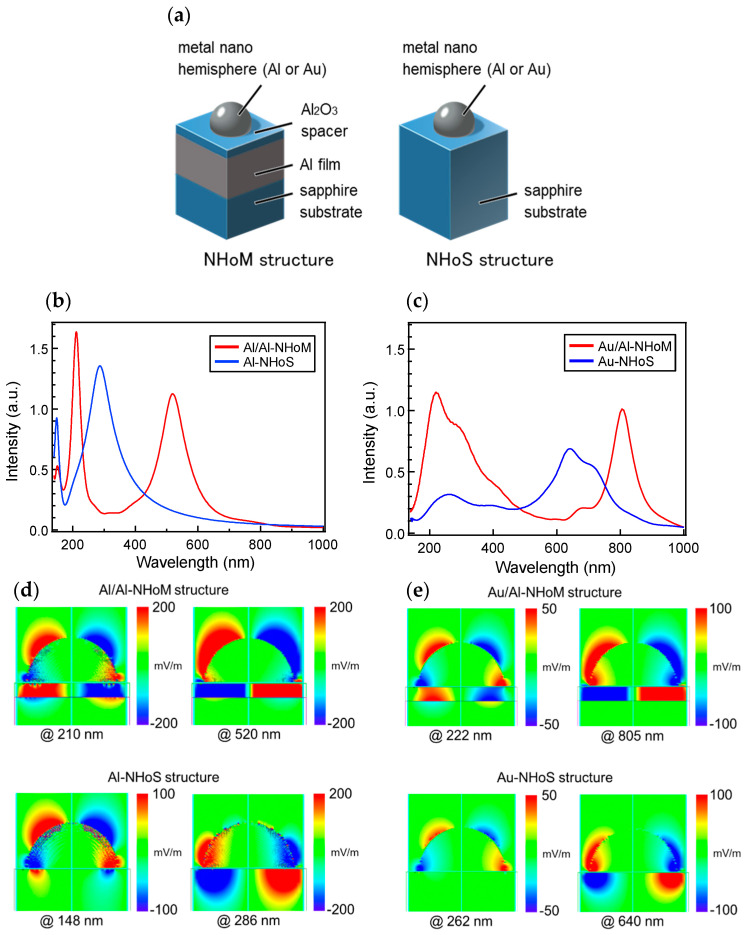
(**a**) Schematic model of the NHoM and NHoS structure; (**b**) calculated extinction spectra for the Al/Al-NHoM and Al-NHoS structure; (**c**) calculated extinction spectra for the Au/Al-NHoM and Au-NHoS structure; (**d**) spatial distribution of electric field (Ez) around the Al/Al-NHoM and Al-NHoS structure; (**e**) spatial distribution of electric field (Ez) around the Au/Al-NHoM and Au-NHoS structure.

Figure 2a illustrates schematic diagrams of the NHoM structure and the Nano-Hemisphere on Sapphire (NHoS) structure. Figure 2b shows the calculated extinction spectra for the homogeneous Al/Al-NHoM structure, which consists of an Al nano-hemisphere on an Al film, and the Al-NHoS structure, featuring an Al hemisphere on a sapphire substrate. Figure 2c displays the calculated extinction spectra for the heterogeneous Au/Al-NHoM structure, composed of an Au nano-hemisphere with an Al film, and the Au-NHoS structure, which consists of Au hemispheres on a sapphire substrate. The diameter of the metal hemisphere was set to 70 nm, and the thickness of the Al_2_O_3_ spacer was set to 11 nm.

For the Al-NHoS structure, two distinct resonance peaks are observed at wavelengths of approximately 150 nm and 290 nm. In contrast, for the Al/Al-NHoM structure, both resonance peaks shift to longer wavelengths, appearing at 210 nm and 520 nm, respectively. Additionally, the peak on the shorter-wavelength side exhibits further sharpening. In the Au-NHoS structure shown in Figure 2c, the LSPR peak for Au appears near 700 nm, accompanied by a smaller peak around 300 nm. The peak near 300 nm originates from the damping effect of the 1020 THz Lorentz term in the Au model, rather than LSPR. In contrast, the Au/Al-NHoM structure exhibits a stronger short-wavelength peak due to the presence of the Al film, although no significant redshift of the peak is observed. The long-wavelength resonance peak in the Au/Al-NHoM structure is sharper and more intense than that in the Au-NHoS structure, with a redshift of approximately 150 nm.

Figure 2d,e show the spatial distribution of the electric field at the peak wavelengths for the Al/Al-NHoM and Al-NHoS structures, as well as the Au/Al-NHoM and Au-NHoS structures, respectively. Each distribution represents the z-component of the electric field (Ez). The electric field distributions at wavelengths of 150 nm and 290 nm for the Al-NHoS structure, as shown in Figure 2d, reveal the generation of quadrupole and dipole vibrational modes within the Al hemisphere [36,40], respectively. When a thick metal layer is introduced beneath the hemisphere to form the Al/Al-NHoM structure, mirror-image modes are induced in the metal film, coupling with the quadrupole and dipole modes in the metal hemispheres. Since the LSPR wavelength in the nano-hemisphere depends on the strength of mode coupling, the resonance wavelength can be controlled by adjusting the thickness of the dielectric spacer [36]. When the vibrational modes of the nano-hemisphere and the metal film couple, the electric field generated by the vibrational mode of the nano-hemisphere and its mirror image mode in the metal film split into two modes: one behaving symmetrically and the other antisymmetrically. In the NHoM structure, where the mirror image in the metal film is utilized, the electric fields of the nano-hemisphere and the metal film are never in phase. Consequently, the peak of the antisymmetrically behaving mode dominates. The antisymmetric coupled modes, in which the electric fields of the nano-hemisphere and film effectively cancel each other out, behave as dark modes in the overall structure, exhibiting strong, sharp peaks. However, due to inherent structural asymmetry, the electric fields do not completely cancel, allowing for interaction with light.

For the Au/Al-NHoM structure, the mechanisms contributing to the enhancement of each peak are different. As shown in Figure 2e, the electric field distribution at 222 nm resembles a quadrupole mode. However, Au does not exhibit SPR at this wavelength. Similar to the Ga_2_O_3_/Al NHoM structures [39], the Au nano-hemispheres localize light, inducing an LSPR in the Al layer. Therefore, the peak at 222 nm is attributed to the LSPR of Al induced by the Au nano-hemisphere, rather than to coupled modes observed in the Al/Al-NHoM structure. In this case, because Al does not form a nanostructure, it does not strictly exhibit LSPR. However, since it can be considered a pseudo-LSPR of Al, it is referred to simply as LSPR. The peak at 222 nm is not due to the interaction between the metal hemisphere and the metal film, which explains the absence of a redshift in the peak. At a wavelength of 222 nm, although LSPR does not occur in the Au nanospheres themselves, the Au nano-hemispheres can generate a peak similar to LSPR, which is a distinctive feature of the heterometallic NHoM structure. At 805 nm, the electric field distribution exhibits dipole modes forming near the equator of the Au hemisphere. The Al film acts as a mirror, producing mirror-image modes that interact with the vibrational modes of the Au nano-hemisphere. This interaction sharpens and redshifts the peak compared to the Au-NHoS structure.

**Figure 3:**
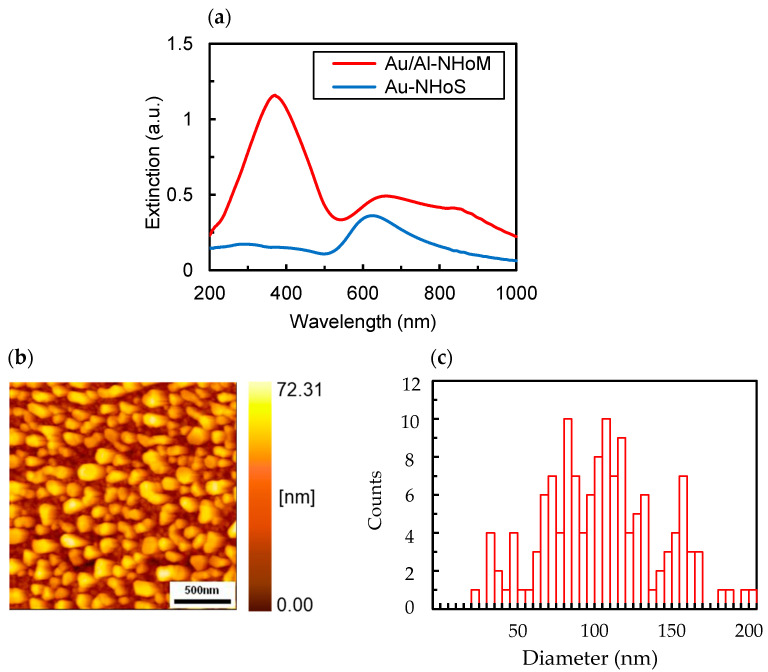
(**a**) Experimental extinction spectra of the Au/Al-NHoM and Au-NHoS structures; (**b**) AFM images of the Au/Al-NHoM structure; (**c**) particle size distribution derived from the AFM image of the Au/Al-NHoM structure.

In this study, Au/Al-NHoM structures were fabricated because Al exhibits high wettability and strong adhesion to substrates, making it challenging to fabricate nano-hemispherical structures with well-defined shapes or large sizes. Figure 3 presents the extinction spectra for the NHoM and NHoS structures obtained from actual samples (a) and an AFM image of the NHoM structure surface (b). The Al_2_O_3_ spacer thickness was set to 10 nm, accounting for the formation of a natural oxide layer, and the Au nano-hemispheres were formed by annealing a 6 nm thick Au film after deposition. In the NHoS structure, a peak at 622 nm is observed on the long-wavelength side, attributed to the Au nano-hemispheres. In the NHoM structure, this long-wavelength peak shifts to 660 nm, and a strong short-wavelength peak at 371 nm, consistent with the simulation results, is newly observed. The long-wavelength peak in the experimental results appears slightly broader compared to the simulations. As shown in Figure 3b, the fabrication of nano-hemispheres by annealing a metal thin film results in variations in the size, shape, and arrangement of nano-hemispheres. Figure 3c displays the results of grain size analysis based on the AFM image in Figure 3b. The sizes of Au nano-hemisphere range from 20 nm to 210 nm, and this variation is likely the primary cause of the broadening observed in the peaks at longer wavelengths.

**Figure 4:**
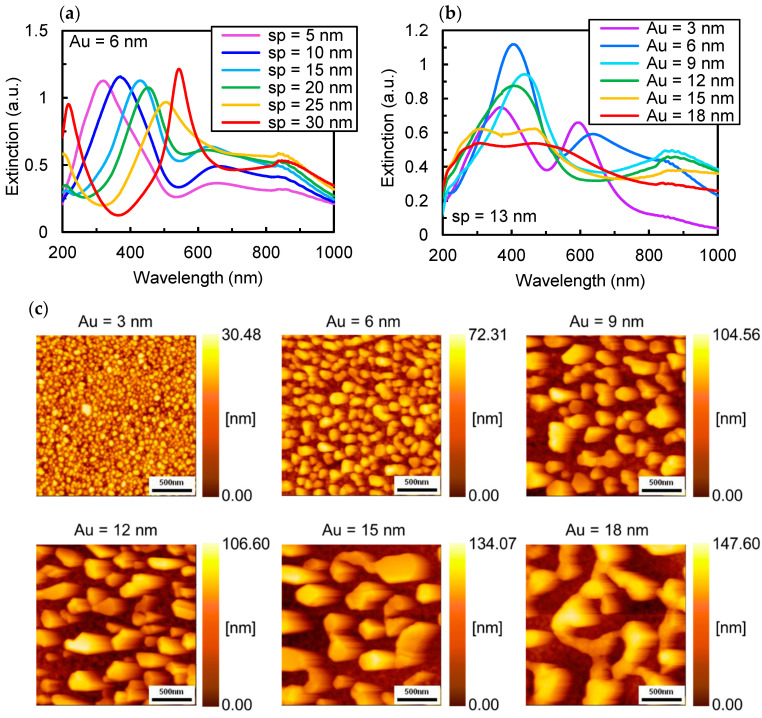
(**a**) Experimental extinction spectra of the Au/Al-NHoM structure with spacer thickness of 5, 10, 15, 20, 25 and 30 nm; (**b**) experimental extinction spectra of the Au/Al-NHoM structure with initial Au film thickness of 3, 6, 9, 12, 15 and 18 nm; (**c**) AFM images of the Au/Al-NHoM structure with initial Au film thickness of 3, 6, 9, 12, 15 and 18 nm.

To evaluate the tunability of the LSPR peak, the thickness dependence of the extinction spectra for the Au thin film and Al_2_O_3_ spacer was investigated. Figure 4a shows the extinction spectra of the NHoM structure, where the Al_2_O_3_ spacer thickness was varied from 5 nm to 30 nm in 5 nm increments. The Au nano-hemispheres were formed by annealing a 6 nm-thick Au thin film deposited on the spacer. As illustrated in Figure 4a, the wavelength of the LSPR peak exhibited a redshift with increasing spacer layer thickness. For a spacer thickness of 5 nm, the peak wavelength is observed at 330 nm, while it shifts to 550 nm for a thickness of 30 nm. This demonstrates that the wavelength can be tuned across a range of 200 nm by simply adjusting the spacer layer thickness. Since the LSPR of Al in the UV region is not due to electric field coupling between the nano-hemisphere and the metal film, this redshift is attributed to an increase in the effective refractive index of the entire structure. The long-wavelength peak exhibits a blueshift as the spacer thickness increases and eventually merges with the short-wavelength peak at spacer thicknesses of 25 nm and 30 nm. As the spacer layer thickness increases, the interaction between the vibrational modes of the Au nano-hemispheres and mirror mode in the Al layer weakens and approaches that of the NHoS structure, causing the resonance peak to shift toward the short-wavelength side.

Figure 4b shows the extinction spectra of the NHoM structure, where the nano-hemisphere structure was formed by heat treatment of Au films with initial thickness varying from 3 nm to 18 nm in 3 nm increments. Figure 4c presents the corresponding AFM images of the surface. The Al_2_O_3_ spacer layer thickness was fixed at 13 nm. A redshift in the resonance peak is observed as the initial Au film thickness increases from 3 nm to 9 nm. As shown in Figure 4c, the thicker Au films result in larger Au nano-hemispheres after annealing. This increase in size likely causes a retardation effect on the vibrational mode induced in the Al substrate, shifting the resonance wavelength toward longer wavelengths. The measured grain sizes and standard deviations are 41.91 ± 18.55 nm for a 3 nm initial film, 104.88 ± 35.54 nm for a 6 nm film, and 174.34 ± 75.58 nm for a 9 nm film. For initial Au film thicknesses up to 9 nm, the grain size could be consistently controlled, despite some variation. For initial thicknesses of 15 nm or more, broadening of the short-wavelength peak is observed. This broadening is likely due to increased grain size variation and the fusion of nanostructures into irregular, amoeba-like shapes, rather than the formation of discrete, independent islands, even after heat treatment.

These findings indicate that controlling the resonance wavelength through the initial thickness of the Au film is limited to a narrow range due to the difficulty in controlling Au nanosphere sizes beyond a certain threshold. In contrast, varying the thickness of the Al_2_O_3_ spacer allows for broader control of peak wavelengths, spanning from the UV to the visible region.

**Figure 5:**
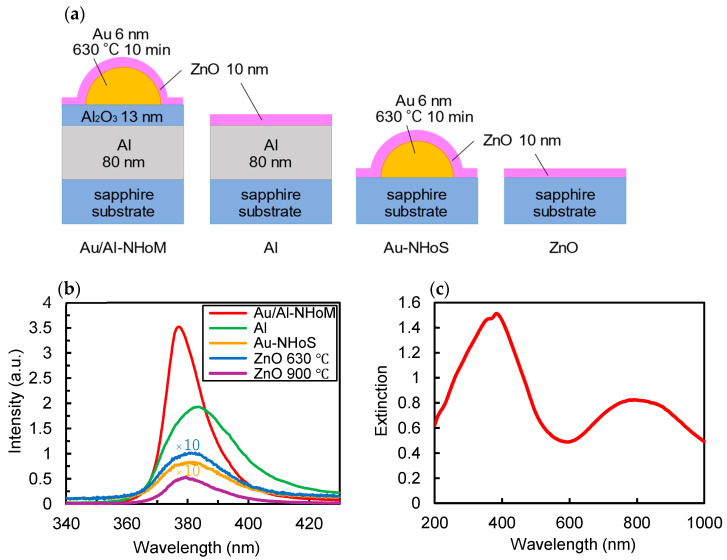
(**a**) Schematic model of ZnO film on the Au/Al-NHoM structure, Al film, Au-NHoS structure, and sapphire substrate; (**b**) PL spectra of ZnO film on the Au/Al-NHoM structure, Al film, Au-NHoS structure, and sapphire substrate; (**c**) extinction spectra of Au/Al-NHoM structure with ZnO film.

To investigate the luminescence enhancement properties, a 10 nm ZnO film was deposited on the NHoM structure using an RF sputtering system. The NHoM structure was prepared by depositing an 80 nm Al film, a 13 nm Al_2_O_3_ spacer layer, and a 6 nm Au layer on a sapphire substrate, followed by annealing at 630 °C for 10 min in a nitrogen atmosphere. For comparison, ZnO was also deposited on the Al layer, the Au-NHoS structure, and the sapphire substrate. Since the sputtered ZnO thin films were amorphous, annealing was applied to improve their crystallinity and achieve band-edge emission. The ZnO film deposited on the sapphire substrate was annealed at 630 °C and 900 °C, while all other samples were annealed at 630 °C. Figure 5a,b show schematic side views and the emission spectra of each sample. Figure 5c shows the extinction spectrum of the NHoM structure with a ZnO film. As shown in Figure 5c, the resonance peak of the NHoM structure with the ZnO film is nearly coincident with the emission wavelength of ZnO at 380 nm. The extinction spectrum of the Au-NHoS structure is similar to that in Figure 3, with no significant resonance peak at 380 nm. A 35-fold increase in peak intensity is observed for the Au/Al-NHoM structure (red line) compared to the ZnO thin film on the sapphire substrate annealed at 630 °C (blue line), demonstrating significant emission enhancement. Even with only the Al layer, a 17-fold enhancement is observed (green line), albeit with a broad peak, likely because only the propagating SPR contributes to the luminescence enhancement in the case of the Al layer. In contrast, no enhancement is observed for the sample with only Au hemispheres (yellow line), and the intensity decreases instead. This reduction occurs because Au behaves as a dielectric in the UV region and absorbs UV light. These results indicate that both the Au hemispheres and the Al film are essential for achieving high luminescence enhancement and effectively controlling the enhancement wavelength.

The PL intensity of the ZnO thin film annealed at 900 °C (purple line) is five times higher than that of the sample annealed at 630 °C. This increase is attributed to improved crystallinity at higher annealing temperatures, leading to stronger band-edge emissions. However, since the melting point of Al is 660 °C, annealing ZnO thin films on NHoM structures above this temperature would cause structural degradation. Therefore, the annealing temperature for the ZnO thin film on the NHoM structure was limited to 630 °C. Despite the lower annealing temperature, the ZnO thin film on the NHoM structure achieves higher luminescence intensity compared to that of the ZnO thin film annealed at 900 °C, demonstrating a significant enhancement in UV light emission enabled by the NHoM structure. If the NHoM structure can withstand annealing at 900 °C while maintaining its shape, even higher PL intensity is expected.

**Figure 6:**
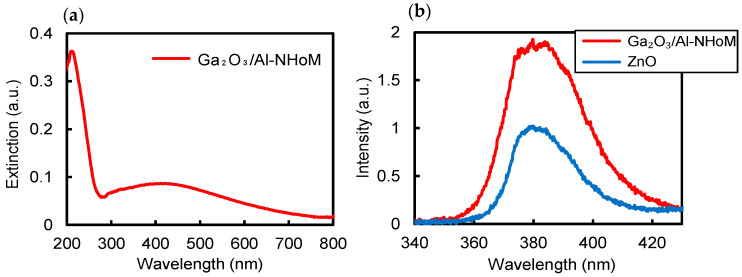
(**a**) Extinction spectra of the Ga_2_O_3_/Al-NHoM structure; (**b**) PL spectra of ZnO film on the Ga_2_O_3_/Al-NHoM structure and sapphire substrate.

For comparison, the extinction spectrum of the Ga_2_O_3_/Al-NHoM structure and the PL spectrum of the ZnO thin film deposited on the Ga_2_O_3_/Al-NHoM structure are presented in Figure 6a,b. The Ga_2_O_3_/Al-NHoM structure was fabricated by sequentially depositing a 100 nm Al film, a 13 nm Al_2_O_3_ spacer layer, and a 15 nm Ga layer on a sapphire substrate, followed by annealing at 300 °C for 10 min in a nitrogen atmosphere to form Ga nano-hemispheres. The structure was then oxidized by annealing at 500 °C for 10 min in air, yielding the Ga_2_O_3_/Al-NHoM structure. The extinction spectra reveal a resonance peak at approximately 200 nm, which is blue-shifted compared to the Au/Al-NHoM structure. PL measurements reveal that the emission intensity of the ZnO thin film on the Ga_2_O_3_/Al-NHoM structure is approximately 1.8 times higher than that on the sapphire substrate. The Ga_2_O_3_/Al-NHoM structure was fabricated with a spacer layer thickness of 13 nm to shift the resonance peak and overlap it with the ZnO emission. However, the resonance peak remained around 200 nm, indicating that it is challenging to achieve the resonance at the ZnO emission wavelength. This explains the lower enhancement, suggesting the advantage of using Au nano-hemispheres over Ga_2_O_3_ nano-hemispheres to enhance the luminescence of ZnO thin films.

**Figure 7:**
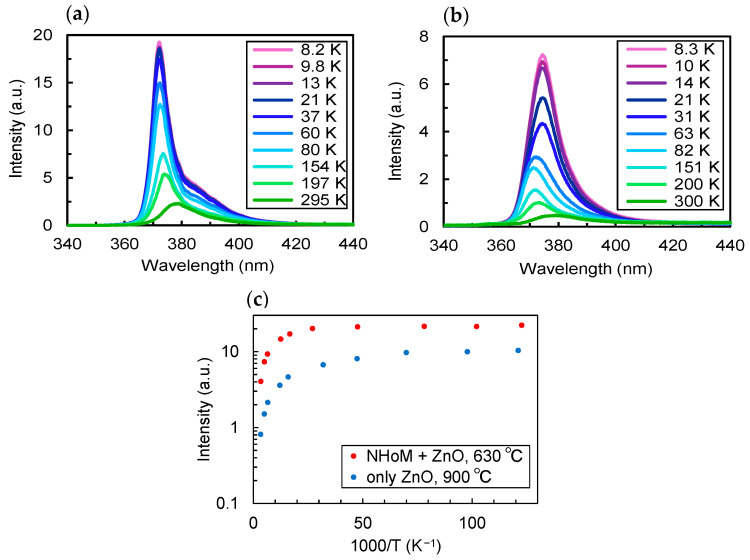
(**a**) PL spectra of ZnO film on the Au/Al-NHoM structure at room to low temperatures; (**b**) PL spectra of ZnO film on sapphire substrate at room to low temperatures; (**c**) temperature dependence of the luminescence intensity for the ZnO thin film on the Au/Al-NHoM structure and the sapphire substrate.

To estimate the internal quantum efficiency (IQE), we measured the temperature dependence of the band-edge emission from the ZnO thin film. Figure 7a presents the temperature-dependent PL spectrum of the ZnO thin film on the NHoM structure and annealed at 630 °C, while Figure 7b shows the PL spectrum of the ZnO thin film on a sapphire substrate and annealed at 900 °C. The full width at half maximum (FWHM) of the PL spectrum of the ZnO thin film on the NHoM structure is 14 nm at room temperature and 7 nm at cryogenic temperature, while the FWHM on the sapphire substrate is 24 nm at room temperature and 12 nm at cryogenic temperature. At room temperature, the FWHM of the NHoM structure is narrower than ZnO thin film only, which can be attributed to the LSPR effect in the NHoM structure that enhances and sharpens the photoluminescence spectrum.

Arrhenius plots of the area intensities, calculated from the spectra in Figure 7a,b, are presented in Figure 7c. The luminescence intensity of ZnO on the NHoM structure becomes nearly saturated in the low-temperature region, where the IQE approaches 100%. Assuming IQE at cryogenic temperatures is 100%, we estimated the room-temperature IQE based on the ratio of the PL area intensities at room and cryogenic temperatures. For the ZnO thin film on the NHoM structure, the PL area intensity is 22.22 at cryogenic temperature and 4.04 at room temperature, resulting in an estimated IQE of 18%. In comparison, the ZnO thin film on the sapphire substrate has a PL area intensity of 10.35 at cryogenic temperature and 0.81 at room temperature, yielding an estimated IQE of 8%. The PL intensity of the ZnO thin film on the sapphire substrate did not saturate even at 8 K, suggesting that its IQE may not have reached 100% at this temperature. Therefore, the actual IQE could be even lower if measured at lower temperatures. These results indicate that integrating ZnO thin films with the NHoM structure improves the internal quantum efficiency of band-edge emission by at least a factor of two. Although the ZnO thin film annealed at 900 °C on a sapphire substrate exhibits five times higher luminescence intensity than the 630 °C-annealed film, the ZnO thin film on the NHoM structure annealed at 630 °C surpasses in terms of luminescence intensity, FWHM, and IQE at room temperature. This enhancement in IQE can be attributed to the reduction in non-radiative recombination through SPR. In the absence of metal structures, electrons and holes generated by photoexcitation undergo either radiative or non-radiative recombination. However, in the presence of metallic nanostructures, if the energy of the electron–hole pair matches the electronic vibration energy of the surface plasmon, they combine and are emitted as light. The emission process through SPR is significantly faster than other processes, which improves IQE by suppressing slower non-radiative recombination.

Furthermore, Au exhibits excellent chemical stability, while the Al layer demonstrates strong resistance to oxidation due to the protective spacer layer. This makes the Au/Al-NHoM structure highly resistant to oxidation, ensuring its long-term stability.

Recent studies have demonstrated that breaking time-reversal symmetry (TRS) in plasmonic and metamaterial structures can lead to nonreciprocal scattering effects, which have been explored using gyromagnetic and gyroelectric materials [41,42,43]. By replacing the metallic hemisphere with gyrotropic materials, it may be possible to achieve controllable nonreciprocal LSPR enhancement, which could further optimize light extraction efficiency in ZnO-based optoelectronic devices.

## 4. Conclusions

We reported the optical properties of heterometallic NHoM structures composed of Au nano-hemispheres and Al films. The Au/Al-NHoM structure fabricated in this study can be produced over large areas in a short time using simple methods such as evaporation, sputtering, and annealing, without the need for complex nanofabrication techniques like electron beam lithography. The Au/Al-NHoM structure exhibited sharper and more distinct LSPR peaks compared to conventional Al-only nanostructures. Additionally, by adjusting the thickness of the Al_2_O_3_ spacer layer, the resonance wavelength can be tuned across a broad range from the UV to the visible region. The combination of the NHoM structure with a sputter-deposited ZnO thin film achieved a remarkable 35-fold enhancement in UV luminescence intensity. Furthermore, by examining the temperature dependence of the luminescence intensity, we estimated the internal quantum efficiency (IQE), which was found to have improved by at least a factor of two. Controlling the emission enhancement wavelength of the NHoM structure is expected to improve the emission efficiency of light-emitting devices across a broad range of wavelengths. Specifically, the hetero-metallic NHoM structure utilizing Al is particularly effective for achieving high-efficiency emission in the deep UV wavelength range, where luminescence efficiency is typically low. We propose that combining NHoM structures with ultra-wide-bandgap semiconductors, such as oxide semiconductors, provides an effective pathway for realizing high-efficiency deep-UV LEDs. Unlike AlGaN, which is primarily used for deep-UV light emission, oxide semiconductors can be fabricated using simple processes like spin-coating. When integrated with the proposed NHoM structure, this combination enables the fabrication of high-efficiency deep-UV LEDs through straightforward and cost-effective methods. Furthermore, by designing an LED structure that positions the NHoM structure close to the emission layer, it becomes possible to create next-generation high-efficiency deep-UV LEDs that emit light via current injection.

## Data Availability

The data presented in this study are available from the corresponding author upon request.
